# Advances in the Knowledge of the Underlying Airway Remodeling Mechanisms in Chronic Rhinosinusitis Based on the Endotypes: A Review

**DOI:** 10.3390/ijms22020910

**Published:** 2021-01-18

**Authors:** Kijeong Lee, Junhu Tai, Sang Hag Lee, Tae Hoon Kim

**Affiliations:** Department of Otorhinolaryngology-Head & Neck Surgery, College of Medicine, Korea University, Seoul 02841, Korea; peppermint_1111@hotmail.com (K.L.); junhu69@korea.ac.kr (J.T.); sanghag@kumc.or.kr (S.H.L.)

**Keywords:** chronic rhinosinusitis, tissue remodeling, endotypes, airway remodeling

## Abstract

Chronic rhinosinusitis (CRS) is a chronic inflammatory condition of the nasal and paranasal sinus mucosa that affects up to 10% of the population worldwide. CRS is the most representative disease of the upper respiratory tract where airway remodeling occurs, including epithelial damage, thickening of the basement membrane, fibrosis, goblet cell hyperplasia, subepithelial edema, and osteitis. CRS is divided into two phenotypes according to the presence or absence of nasal polyps: CRS with nasal polyp (CRSwNP) and CRS without nasal polyps (CRSsNP). Based on the underlying pathophysiologic mechanism, CRS is also classified as eosinophilic CRS and non-eosinophilic CRS, owing to Type 2 T helper (Th2)-based inflammation and Type 1 T helper (Th1)/Type 17 T helper (Th17) skewed immune response, respectively. Differences in tissue remodeling in CRS are suggested to be based on the clinical phenotype and endotypes; this is because fibrosis is prominent in CRSsNP, whereas edematous changes occur in CRSwNP, especially in the eosinophilic type. This review aims to summarize the latest information on the different mechanisms of airway remodeling in CRS according to distinct endotypes.

## 1. Introduction

Chronic rhinosinusitis (CRS) is defined as a chronic inflammatory condition of the nasal and paranasal sinus mucosa, which affects approximately 12% of the population worldwide [1]. CRS is classified into the following two phenotypes based on the presence or absence of nasal polyps using endoscopic examination or computed tomographic findings: CRS with nasal polyps (CRSwNP) and CRS without nasal polyps (CRSsNP) [2,3]. Owing to the pathogenesis of CRS, there has been recent emphasis on the importance of classifying CRS based on the endotypes [4,5]. Previously, depending on the phenotype, CRSsNP was known to exhibit neutrophil dominance, whereas CRSwNP is an eosinophil-dominant type 2 inflammation [6]. However, unlike American and European patients, most Asian CRSwNP patients display neutrophil dominance, which accounts for more than 50% of cases. CRSsNP is reported to exhibit a type 2-biased immune response as well as type 1 or type 3 inflammation [7,8,9]. In addition, it has been identified that type 2 immune response is present in CRSsNP as well [10]. Thus, both phenotypes are presently further classified according to the endotypes, based on immune response characterization. In the European Position Paper on Rhinosinusitis and Nasal Polyps (EPOS) 2020, the classification of CRS was defined based on anatomic distribution (localized vs. diffuse) and endotype dominance (type 2 vs. non-type 2) rather than the presence of nasal polyp [1].

Tissue remodeling is defined as the abnormal reconstruction or restitution of the damaged tissue in relation to inflammation or mechanical injury that occurs in any organ. In lower airway diseases, including asthma, chronic obstructive pulmonary disease, and bronchiectasis, the concept of tissue remodeling has been studied in detail; however, only a few studies in relation to the upper airway have been carried out [11]. In asthma, airway remodeling, including goblet cell hyperplasia, subepithelial collagen deposition, thickened basement membrane, increased airway smooth muscle, and angiogenesis is affected by several underlying mechanisms and influences disease prognosis [12]. Allergic rhinitis (AR) and CRS are representative upper airway diseases known to be strongly related to asthma. In AR, tissue remodeling is not a representative feature, and the evidence related to remodeling is conflicting [13]. However, in CRS, tissue remodeling is a prominent feature.

In CRS, tissue remodeling has been reported to include mucosal hypertrophy, basement membrane thickening (BMT), fibrosis, collagen deposition, angiogenesis, and osteitis [14]. However, although classification based on the endotype of the disease is considered important, there is no literature review that summarizes the differences in remodeling patterns. Here, we sought to describe the recent perceptions with regards to the end of CRS and features of the upper airway remodeling according to each endotype.

## 2. Current Perceptions in CRS Classification

As the importance of precision medicine is emphasized in all medical fields for CRS, it is considered important to divide the spectrum of disease based on the underlying pathomechanism. Immune response is generally categorized into three inflammatory endotypes according to the inflammatory mediators, infiltrating immune cells, and physiologic functions. A distinct subset of CD4+ T cells [T helper (Th)1, Th2, and Th17 cells) plays important roles in the induction of a distinct inflammatory process according to the endotypes [15]. Based on the infiltrating cell types, CRS was initially classified as eosinophilic and non-eosinophilic CRS. Eosinophilic CRS, which is dominant in American and European patients, presents a type 2 immune response, whereas non-eosinophilic CRS, a neutrophil-dominant type prominent in Asian patients, causes either type 1 or type 3 inflammation [16]. Recent studies, including cluster analysis and multi-regional studies, further subdivided the endotypes and suggested that several endotypes could be present in one patient [17,18,19]. Nevertheless, the most fundamental classification for the endotype of CRS is type 2 or non-type 2. In this section, we summarize the pathogenesis of CRS according to either type 2 CRS or non-type 2 CRS, which leads to different clinical features and distinct upper airway remodeling patterns (Table 1) [20,21].

### 2.1. Type 2 Chronic Rhinosinusitis

Type 2 CRS is characterized by eosinophil-dominant inflammation. Type 2 immune response is triggered by foreign matter stimulation of the nasal epithelium following secretion of thymic stromal lymphopoietin (TSLP), interleukin (IL)-25, IL-1, and IL-33 from epithelial cells [22,23]. TLSP stimulates and activates myeloid dendritic cells (DCs) to present antigen and co-stimulatory signals, thereby differentiating naïve T cells into effector Th2 cells; maturation subsequently occurs under an IL-4 environment, and Th2 cells secrete type 2 cytokines, including IL-4, IL-5, and IL-13, which is a hallmark of type 2 inflammation [24]. TSLP and IL-33 also enhance type 2 cytokine production by stimulating type 2 innate lymphoid cells (ILC2s) [23]. TSLP, IL-33, and IL-1 stimulate mast cells in the nasal mucosa for IL-5 and IL-13 secretion [25].

Antigen and co-stimulatory signals present on Th2 cells induce antibody production in B cells. Furthermore, IL-4 secreted from Th2 cells, dendritic cells, and ILC2s trigger antibody isotype switching to immunoglobulin E (IgE) [26]. IL-5 is known to be a key factor in the recruitment and activation of eosinophils, inducing the production of eosinophilic extracellular traps [27]. In addition, Chemokine (C-C motif) ligand (CCL) 23 produced from eosinophils via IL-5 stimulation recruit macrophages and their differentiation into M2 macrophages, which release eotaxins and CCL18 to recruit eosinophils, Th2 cells, and myeloid DCs [28,29].

The production of *Staphylococcus aureus (S. aureus)* enterotoxin, which acts as a super antigen either via direct interaction with MHC cells II on T cells or through binding to the TCRβ-chain, is reported to activate T cells [30]. *S. aureus* enterotoxin also induces the release of Th2 cytokines and enhances eosinophilic inflammation, even independently of enterotoxin activities, by inducing the secretion of IL-33 and TSLP from epithelial cells [31].

### 2.2. Non-Type 2 Chronic Rhinosinusitis

Non-type 2 CRS is characterized by neutrophilic inflammation in the nasal mucosa, which is triggered by infections or external irritants, such as air pollutants. Th1 cell-based type 1 inflammation and Th17 cell-based type 3 inflammation are mixed in non-type 2 CRS. External pathogen invasion induces the production of IL-6, IL-8, and tumor necrosis factor α (TNF-α) from the nasal epithelium [32]. In addition, the interaction between pathogens and Pathogen-associated molecular pattern/Toll-like receptor (PAMP/TLR) stimulates the production of interferon-γ (IFN-γ) and IL-8 [32]. These cytokines and chemokines activate DCs to differentiate CD4-positive T cells into effector T cells. IFN-γ secretion from epithelial cells after pathogen recognition induces Th1 differentiation, which is followed by the secretion of IFN-γ and IL-2 from Th1 cells, leading to type 1 inflammation [32]. Th17 and Th22 cell differentiation occurs in response to the release of IL-6 from epithelial cells, leading to type 3 inflammation. Th17 cells secrete IL-17 and IL-22, whereas Th22 cells only produce IL-22 [33]. IL-8 secreted from epithelial cells recruits neutrophils, which release inflammatory cytokines, such as IL-1β, IL-6, and IL-18, as well as myeloperoxidase (MPO) [34].

Transforming growth factor-β (TGF-β) stimulation induces regulatory T (Treg) cell differentiation from naïve T cells. Furthermore, in response to the secretion of IL-2 from Th1 cells, Treg maturation occurs [35]. Tregs are key regulators of the immune response as they produce the anti-inflammatory cytokine, IL-10, and reduce the function of Th1 and Th2 [36]. Tregs also secrete TGF-β, which is known to play an important role in tissue remodeling in non-type 2 CRS [37].

## 3. Tissue Remodeling

Tissue remodeling in CRS is a dynamic process that involves different temporary or permanent changes. Traditionally, differences in remodeling patterns according to the presence of nasal polyps have been reported. In CRSwNP, remodeling is characterized by pseudocyst formation, stromal edema, reduced collagen deposition, and inflammatory cell infiltration [38]. Notably, eosinophilic CRSwNP is known to be more edematous and less fibrotic than non-eosinophilic CRSwNP [20,39]. However, remodeling in CRSsNP is characterized by BMT, fibrosis, and goblet cell hyperplasia, ultimately presenting TGF-β as a critical mediator [40]. In this section, we discuss the pathogenesis of tissue remodeling in CRS and the associated inflammatory type for the induction of epithelial, mucosal, and bone remodeling.

### 3.1. Epithelial Remodeling

The epithelium serves as the primary barrier for physical, chemical, and immunologic stimuli, ultimately protecting the host from the external environment [41]. Respiratory epithelial cells consist of different cell types [42]. Common cell types include ciliated cells, basal cells, club cells, and mucous-secreting goblet cells, whereas rare cell types include neuroendocrine cells, ionocytes, and solitary chemosensory cells. In the airway, damaged epithelium plays a key role in driving tissue remodeling [43]. Epithelial damage has been considered an important feature owing to bronchoconstriction and ongoing inflammation in lower airway diseases, such as asthma. Epithelial damage in CRS has also been studied previously [44]. Goblet cell hyperplasia and excessive mucin production are important features of CRS. The expression of Mucin 5AC (MUC5AC) and Mucin 5B (MUC5B) were significantly upregulated in the sinus mucosa harvested from CRS patients compared to those from the control [45]. Previous studies identified that MUC5AC played an important role in airway inflammation.

Epithelial–mesenchymal transition (EMT) is defined as the transformation of a polarized epithelial cell into a mesenchymal cell phenotype, inducing loss of typical morphology of each epithelial cell, cell-to-cell junction, and polarity [46]. EMT is known to cause pathological remodeling in lower airway diseases and cancer progression. In CRS, EMT is also considered to be associated with disease recalcitrance [37,47]. During EMT, epithelial markers, such as E-cadherin and sirtuin 1 (SIRT1), are downregulated whereas mesenchymal markers, including N-cadherin, fibronectin, vimentin, alpha-smooth muscle actin (α-SMA), and matrix metalloproteinase (MMP), are upregulated [48]. In this section, we outline the epithelial remodeling in CRS and the associated inflammatory changes.

**Figure 1:**
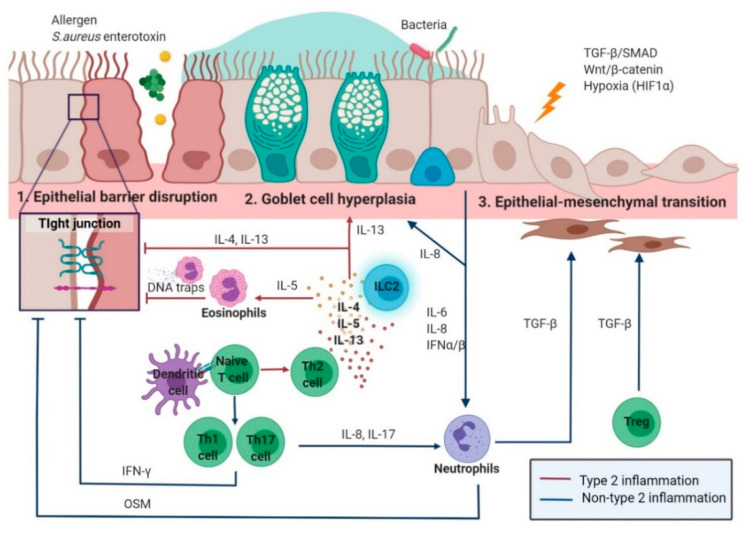
Pathophysiology of epithelial remodeling (epithelial barrier disruption, goblet cell hyperplasia, and epithelial–mesenchymal transition) in chronic rhinosinusitis according to the type of inflammation (type 2 versus non-type 2) (figure created using Biorender.com).

The graphical summary of epithelial remodeling in CRS is shown in Figure 1. Section 3.1.1 and Section 3.1.2 introduce the effect of inflammation on epithelial remodeling based on the type of immune response (Type 2 versus none-type 2) in CRS.

#### 3.1.1. Type 2 Inflammation in Epithelial Remodeling

Previous studies have attempted to identify the correlation between epithelial disruption and eosinophil infiltration. Ponikau et al. reported that all histologic specimens obtained from 22 patients with refractory CRS had epithelial damage [49]. In the same study, eosinophil infiltration in CRS specimens was also identified; however, the relationship between the degree of epithelial damage and eosinophil infiltration was not assessed. The significant correlation between epithelial damage in eosinophil infiltration was first reported by Saitoh et al., suggesting that eosinophils migrating to the epithelium secrete inflammatory mediators, aggravating epithelial damage [50]. Recently, extracellular eosinophilic trap, a complex meshwork of granule protein DNA fibers produced from eosinophils, has been suggested to be associated with epithelial barrier defects in the nasal mucosa of CRS patients [51].

The effect of cytokines on epithelial integrity in CRS pathogenesis has been evaluated. Moreover, Soyka et al. reported, using air–liquid interface culture of epithelial cells from patients with CRSwNP, that stimulation of IFN-γ and IL-4 diminished epithelial barrier function, whereas IL-17 had no influence on transepithelial resistance [52]. A recent experimental study using nasal biopsies from patients with CRSwNP and primary human nasal epithelial cells reported that IL-13-matured nasal biopsies or cells showed significantly decreased expression of tight junction proteins. Furthermore, rhinovirus-induced epithelial barrier dysfunction was aggravated by IL-13 stimulation [53]. *S. aureus* enterotoxin B has also been identified as a driving factor for barrier disruption via IL-6 and IL-8 production in nasal epithelial cells harvested from CRSwNP patients [54]. 

Protease inhibitors are known to protect barrier integrity from environmental protease allergen stimulation. Compared to epithelial cells harvested from healthy patients or non-eosinophilic CRS patients, two endogenous protease inhibitors, namely *Serine peptidase inhibitor Kazal Type 5 (SPINK5)* and cystatin A showed decreased expression in nasal epithelial cells retrieved from eosinophilic CRS patients, thereby suggesting the role of endogenous protease inhibitor imbalance on the epithelial barrier in type 2 CRS [55,56]. 

Studies on the regulation of mucin genes in response to cytokines reported that IL-4, IL-13, IL-8 IL-33, and TNF increase the mRNA and protein expression levels of MUC5AC in nasal polyp-derived epithelial cells [57,58]. The increased expression of pendrin, an epithelial anion transporter, was associated with IL-13-induced mucus production in CRSwNP [59].

#### 3.1.2. Non-Type 2 Inflammation in Epithelial Remodeling

TGF-β1 signaling is considered to be a potent stimulator of EMT, promoting the transition of epithelial cells to myofibroblasts. Accordingly, it induces tissue remodeling and the pathogenesis of CRS. TGF-β1 is known to be upregulated in CRSsNP; however, in CRSwNP, it is downregulated [37]. TGF-β1 initially binds to TGF-βR II. Thereafter, TGF-βR II recruits and phosphorylates TGF-βRI, which is followed by the phosphorylation of R-Smad, a complex of Smad2 and Smad3. Phosphorylated R-Smad binds to Smad4, forming Co-Smad, which shuttles into the nucleus to bind the target gene and regulate transcription [60].

Several epigenetic regulations have been reported to be related to TGF-β1-induced EMT. Activation of histone deacetylase (HDAC) 2 and HDAC4 enhances TGF-β1-induced EMT. Inhibitors of these genes suppress the expression of EMT markers in primary nasal epithelial cells [61]. Li et al. also reported that microRNA-21 mediates TGF-β1-induced EMT through the PTEN/Akt pathway in primary nasal epithelial cells of CRSwNP patients [62].

Wnt signaling also upregulates mesenchymal markers, such as α-SMA and vimentin, by increasing β-catenin in CRSwNP [63]. In neutrophil-dominant CRS patients, IFN-γ promotes EMT through pathways, such as the p38 and extracellular signal-regulated kinase (ERK) pathway [64]. Hypoxia was also found to induce EMT via the downregulation of PP2Ac phosphatase and upregulation of pSmad3, contributing to nasal polyposis; this hypoxia-induced EMT was suppressed by hypoxia-inducible factor (HIF)-1α inhibitors [65]. HIF-1α-induced EMT was also inhibited by the activation of SIRT1, a histone deacetylase, in sinonasal specimens harvested from CRS patients as well as in a nasal polypogenesis murine model [66]. HIF-1α was upregulated in CRSsNP and non-eosinophilic CRSwNP compared to that in healthy sinus mucosa and eosinophilic CRSwNP [49].

In nasal epithelial cells harvested from healthy sinus, the stimulation of cytokines, including TGF-β and IFN-β, and neutrophil elastase, also upregulated MUC5AC expression, whereas lipopolysaccharides and platelet activating factor downregulated MUC5AC expression. Among the TGF-β isoforms, TGF-β1 is considered to have the greatest effect on MUC5AC expression via the Smd signal transduction pathway and TGFβ-IIR binding [67]. However, TNF and IL-1β showed conflicting results in normal nasal epithelial cells. The exposed-CRS rabbit model also showed markedly increased MUC5AC expression. IL-17A was reported to induce MUC5AC expression and goblet cell hyperplasia via the act1 signaling pathway in CRSwNP-derived epithelial cells, ultimately suggesting the mechanism of mucin secretion by Th17-dominant sinus inflammation in Asian CRS patients [68]. Neutrophils also induce excessive mucin secretion through pathways, such as the TNF-, TGF-β, epidermal growth factor receptor, and α-convertase. Another study reported that pendrin enhances mucus production by inducing goblet cell hyperplasia and neutrophil infiltration in Chinese patients with CRS. Human neutrophil elastase, a serine protease released from neutrophils, is known to have an effect on goblet cell hyperplasia and excessive mucin secretion in patients with CRS [69,70].

For the epithelial disruption observed in type 1 CRS, Wang et al. reported that TLR2 was highly related to epithelial damage in CRSsNP patients [71]. In the same study, CRSsNP patients had higher expression of Toll-like receptors (TLR)2, TLR4, and TGF-β1 as well as higher collagen deposition than patients with CRSwNP, including neutrophil infiltration. Furthermore, oncostatin M (OSM) produced from neutrophils was highly increased in nasal polyps compared to that in healthy uncinate tissue. Stimulation of OSM was previously found to decrease transepithelial resistance and reduce barrier function in an air–liquid interface culture of nasal epithelial cells.

### 3.2. Subepithelial Remodeling

**Figure 2:**
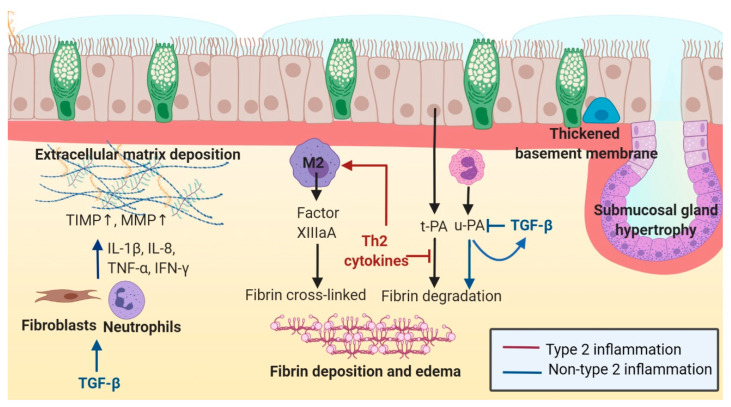
Subepithelial remodeling (extracellular matrix deposition, fibrin deposition, basement membrane thickening, and submucosal hypertrophy) according to the type of inflammation in chronic rhinosinusitis.

Tissue remodeling beneath the epithelium involves BMT, extracellular matrix (ECM) deposition, stromal edema, and glandular hyperplasia (Figure 2). The histology of nasal polyps also includes pseudocyst formation, edematous stroma with albumin deposition, absence of collagen production, and infiltration of inflammatory cells (eosinophils and neutrophils). Studies comparing eosinophilic and non-eosinophilic nasal polyps noted that eosinophilic nasal polyps are often associated with stromal edema and thick BM, whereas non-eosinophilic nasal polyps are related to glandular hypertrophy, fibrosis, and more pseudocysts than eosinophilic nasal polyps.

BMT is known to be the result of collagen deposition, and in CRS, type III and type V collagen are predominant [72]. The duration of CRS symptoms was found to have a positive correlation with BM thickness [73]. Studies comparing the histologies between adult and pediatric CRS patients reported that BMT was more dominant in adult CRS patients, suggesting that the duration of inflammation might be related to BMT [74,75]. 

ECM accumulation is also a critical process in tissue remodeling, leading to either pathologic reconstruction or normal reconstruction [76]. MMPs are the major proteolytic enzymes responsible for extracellular degradation in inflammation, tumor invasion, and chronic degenerative diseases [77]. MMPs recruit immune cells and alter cellular communication as well as immune responses via the proteolytic process of cytokines, chemokines, and complement components and their receptors. The function of MMPs is regulated by tissue inhibitors of metalloproteinases (TIMPs), and the balance between MMPs and TIMPs is considered to play a critical role in the production and degradation of individual components of the ECM, thereby affecting the pathogenesis of airway inflammation [77]. Among the 26 proteins in the MMP family, MMP-9, MMP-2, and MMP-9 have been studied comprehensively in CRS; however, conflicting results have been reported. MMP-9, which mainly degrades gelatin, aggrecan, and elastin, was considered to be associated with nasal polyp formation and TIMP-1 regulated MMP-9 activity [78]. 

Besides goblet cells, submucosal gland hyperplasia and hypertrophy are other sources of mucin overproduction in CRS [79]. Moreover, the submucosal glands have been regarded to play a more important role than goblet cells in mucin gene expression and mucous secretion in CRS patients [80]. In addition, mucus expulsion from the submucosal gland in CRS patients causes decreased mucous strand velocity, resulting in defective mucociliary clearance [81]. A microarray study that investigated differences in the expression of genes associated with submucosal gland hyperplasia between healthy sinus mucosa and remodeled sinus mucosa of CRS patients revealed an increase in the expression of *Dexmoglein 3 (DSG3)* and *Parathyroid hormone like hormone (PTHLH)* in serous cells and *Keratin 14 (KRT14)* in myoepithelial cells of CRS mucosa [82].

In Section 3.2.1 and Section 3.2.2, we describe the type 2 immune pathogenesis and the non-type 2 mechanism in the subepithelial remodeling of CRS, respectively.

#### 3.2.1. Type 2 Inflammation in Subepithelial Remodeling

Previous studies have reported that in nasal polyps, a positive correlation exists between eosinophilic infiltration and BM thickness [50,83]. Saitho et al. identified that this correlation between BMT and eosinophils is stronger for the endothelial eosinophils than eosinophils in the subepithelial layer [50]. In addition, a recent study on the impact of histopathological factors on surgical outcomes reported that thickened BM and mucosal eosinophilia are the main factors that affect the poor prognosis of endoscopic sinus surgery [84].

In an experimental study using organ culture of nasal polyps, *S. aureus* infection promoted the release of MMP-2, MMP-9, TIMP-1, and Th2 cytokines, including IL-5, IL-13, and eotaxin [85]. Another study on the relationship between MMP and cytokines in nasal polyps reported a significant correlation between IL-5 and TIMP-1, suggesting their potential role in the maintenance of homeostasis in nasal polyp [86]. Watelet et al. reported that MMP-9 was increased in both CRSwNP and CRSsNP, whereas MMP-7 was only elevated in CRSwNP compared to that in the controls [87]. The same group suggested that upregulated TIMP-1 in CRSsNP might counteract the activity of MMPs, whereas no definite changes in TIMP-1 activity in CRSwNP could result in edema [88]. However, another study revealed that the increased levels of MMP-2 in nasal polyps elevated MMP-9 expression in CRSsNP, and decreased levels of TIMP-1 in both CRSsNP and CRSwNP were comparable to those in the controls [89].

Tissue edema in nasal polyps was reported to be associated with an imbalance among factors related to coagulation. The increased expression of coagulation factors and decrease in fibrinolytic activity induce fibrin accumulation in the nasal polyp; this is followed by “scaffold” formation, which traps plasma proteins to enhance edema [90]. Coagulation factor XIIIa, which is increased in type 2 nasal polyps, and the tissue plasminogen activator (t-PA) are two factors that are thought to play a role in fibrin accumulation [91]. As Th2 cytokines suppress t-PA in nasal epithelial cells, which play a role in fibrin degradation, fibrin accumulation occurs in a type 2 inflammation environment, thereby leading to edematous eosinophilic nasal polyp [92]. Sejima et al. reported upregulated expression of another fibrinolytic component, urokinase-type plasminogen activator (uPA), in CRSwNP, especially in eosinophils [93]. Their study revealed a significant correlation between uPA and ECP and suggested that excessive uPA expression might interfere with the normal TGF-β activated feedback mechanism of uPA in CRSwNP, resulting in edema in these patients.

In a recent study of mucin secretion in type 2 CRS, the IL-5 positive CRSwNP group showed upregulated expression of IL-4Rα in the submucosal gland as well as the epithelium. Further, MUC5AC and MUC5B secretion were increased following IL-4 and IL-13 stimulation [94].

#### 3.2.2. Non-Type 2 Inflammation in Subepithelial Remodeling

In a study of ECM marker expression in Chinese CRS patients, CRSsNP showed elevated concentrations of TIMP-1, TIMP-4, FOXP3, and collagen deposition compared to those in CRSwNP. The authors claimed that this phenomenon is associated with a relative decrease in TGF-β expression and Treg function in CRSwNP compared to those in CRSsNP, resulting in a lack of collagen deposition in CRSwNP [95]. Recently, another study of CRSsNP in Asians reported that the MMP-9/TIMP-1 ratio had a positive correlation with CRSsNP severity, which is consistent with previous studies on lower respiratory tract disease. Accordingly, this finding suggests the association between MMP-9 and neutrophilic inflammation [96]. Another study from the same group reported that the expression level of MMP-9 was upregulated in non-eosinophilic CRSwNP compared to that in CRSsNP and eosinophilic CRSwNP. However, MMP-1 and MMP-3 showed higher expression levels in the nasal polyps of eosinophilic CRSwNP patients than those with non-eosinophilic nasal polyps [97]. Kostamo et al. reported that MMP-8/TIMP-1 and MMP-9/TIMP-1 ratios are increased in non-eosinophilic CRSwNP but the ratios did not significantly differ in eosinophilic CRSwNP compared to the control [76]. In a study of Chinese CRS, CRSsNP resulted in higher levels of TIMP-1 and TIMP-4 than CRSwNP. However, the levels of MMP-7 and MMP-9 were elevated in both CRSwNP and CRSsNP compared to those in controls [95].

A recent study using transcriptome analysis to assess fibroblasts from CRSsNP compared to those from healthy controls revealed an increase in the expression of the nuclear factor erythroid 2-related factor 3 (NFE2L3), which was known to be upregulated in tissues cultured with Th1 cytokines, such as TNF and IFN-γ [98].

### 3.3. Bone Remodeling

**Figure 3:**
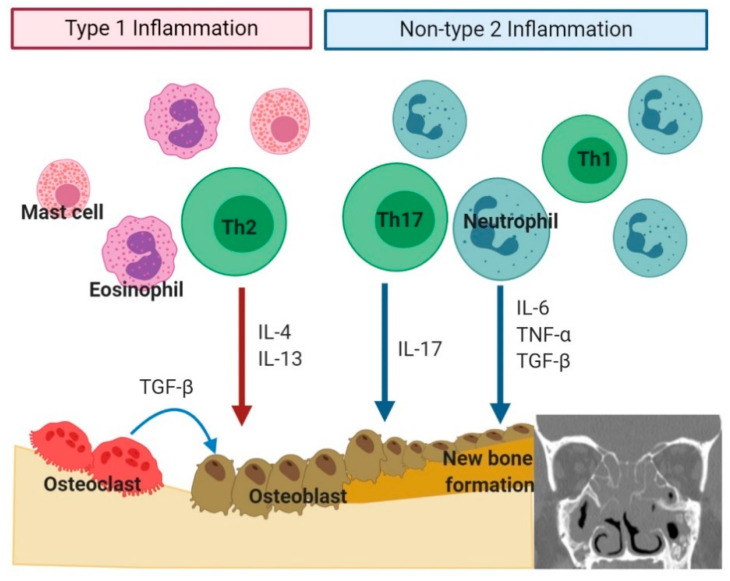
Bone remodeling based on the type of inflammation (type 2 inflammation versus non-type 2 inflammation) in chronic rhinosinusitis (figure created using Biorender.com).

Osteitis, simply an inflammatory bony change, is characterized by neo-osteogenesis with new woven bone formation and periosteal thickening. Based on radiologic or pathologic findings, osteitis is present in 36–53% of CRS patients [99]. Furthermore, it is known to be associated with recalcitrant cases or disease severity [100]. The histological findings of osteitis in CRS include periosteal thickening, new woven bone formation, bone resorption, and fibrosis [101]. The underlying molecular mechanism of osteitis development is not well established. However, there have been attempts to unravel the molecular mechanism of osteitis in CRS using animal models [82]. In the CRS rabbit model, histopathological findings using the sinus bone showed periosteal fibrosis, neo-osteogenesis, and bony degradation in the disease side, affecting the contralateral side as well [102,103]. In Section 3.3.1 and Section 3.3.2, we would discuss the pathogenesis of bone remodeling in CRS based on immune response type (Figure 3).

#### 3.3.1. Type 2 Inflammation in Bone Remodeling

Several studies have investigated the association between eosinophilic inflammation and osteitis in CRS. Snidvongs et al. reported that serum eosinophilia was associated with osteitis in patients, regardless of them having received surgery priorly [100]. Mehta et al. also proposed that a direct correlation exists between blood eosinophil levels and osteitis in relation to sinus mucosal thickening in CRS patients [104]. 

P-glycoprotein (P-gp), an adenosine triphosphate (ATP)-dependent efflux pump expressed on different cell types, is known to be highly expressed in eosinophilic CRS and CRSwNP and is associated with multidrug resistance [105,106]. Gunel et al. reported that a higher expression level of P-gp was found in CRS patients with osteitis than in those without osteitis [107]. In a recent microarray analysis of the genes related to osteitis in CRS, growth differentiation factor 5 and exostosin glycosyltransferase 1 were upregulated in the osteitic bone of CRS patients. Furthermore, they displayed a positive correlation with eosinophilic inflammation [108]. Fibroblast growth factor and colony stimulating factor levels were also increased in osteitic bone [108]. In a murine eosinophilic CRS model induced using *Aspergillus fumigatus*, significant upregulation of bone morphogenetic protein, fibroblast growth factor, and MMP family members was demonstrated [109]. 

The correlation between Th2 cytokines and osteitis was also previously assessed. In an in vitro study using human osteoblasts, IL-4 and Il-13 stimulation induced osteoblast differentiation activity and increased collagen secretion and mineralization [110]. In an experimental study with paranasal mucosa tissue obtained during endoscopic sinus surgery, both the mRNA and protein expression levels of IL-13 were increased in CRS patients with neo-osteogenesis [111]. In the same study, a positive correlation was found between IL-13 and both the Global Osteitis Scoring Scale (GOSS) and in vitro mineralization by osteoblast [111]. In a murine eosinophilic CRS model induced by the administration of ovalbumin and staphylococcal enterotoxin B, histological and radiographic findings of osteitis were identified, thereby demonstrating the positive correlation among bone thickness, Runt-related transcription factor 2 (RUNX2)-immunoreactive osteoblasts, and IL-13 expression [112]. A higher expression level of RUNX2 was observed in osteoblasts harvested from CRS patients with neo-osteogenesis than in those without neo-osteogenesis. Moreover, IL-13 and IL-17 were found to induce osteoblast differentiation via RUNX2 stimulation [113]. 

#### 3.3.2. Non-Type 2 Inflammation in Bone Remodeling

Bacteria-induced osteitis has been reported in CRS. In fact, in experimental studies with a rabbit CRS model, bacterial organisms, including *S. aureus* and *Pseudomonas aeruginosa*, can induce bony changes, such as periosteal thickening, woven bone formation, hyperplastic osteoblasts, osteoclastic activity, and fibrosis [114,115]. A retrospective study of 90 patients undergoing sinus surgery revealed that *P. aeruginosa*, and not *S. aureus*, is an independent predictor of neo-osteogenesis in refractory CRS patients [116]. However, Karampelis et al. reported that neither *P. aeruginosa* nor *S. aureus* was associated with neo-osteogenesis in CRS patients with or without cystic fibrosis [117]. Several studies have also provided evidence of the association between bacterial biofilms and osteitis in CRS patients. In fact, a study with 84 CRS patients reported that CRS patients with higher biofilm volume or biofilm score had higher histopathologic bony grade or greater GOSS score than those with a lower biofilm volume or score [118].

Higher expression levels of inflammatory cytokines, such as IL-6, IL-11, and TNF-α, were identified more in the bone tissue obtained from CRS patients with osteitis than in those without osteitis [119]. TGF-β signaling cascades have also been reported to be involved in the bone remodeling process of both CRSsNP and CRSwNP; this is also accompanied by increased expression of TGF-β and TGF-β receptor 1 as well as a higher number of Smad2/3-positive cells [120].

## 4. Conclusions

Tissue remodeling is a dynamic and complex process. As a key feature of CRS, remodeling involves different layers of sinonasal tissues, including the epithelium, subepithelium, and underlying bone. Tissue remodeling in CRS exhibits distinct clinical features based on the inflammatory pattern of the disease, which is known as “endotype,” and involves different immune cells and inflammatory mediators. In CRS, remodeling is not only a simple consequential change following long periods of chronic inflammation but is also a parallel process that continuously occurs during ongoing inflammation. Remodeling in CRS is known to affect symptom severity, disease prognosis, response to treatment, and recurrence. Thus, to date, different studies have sought to determine whether it is possible to reverse the remodeled tissue in CRS. Steroids, the most widely used medication that exhibits anti-inflammatory activity, could regulate the level of MMPs and TIMPs, induce epithelial repair, and reduce tissue eosinophil infiltration [121,122]. In addition, a remarkably reduced amount of TGF-β1 positive cells and a thinner basement membrane were identified in a steroid-treated CRS group compared to the controls [123]. Doxycycline could have the potential to reduce nasal polyp volume by decreasing MMP-9 levels and reducing MUC5AC levels [124,125]. However, doxycycline did not significantly alter IL-5 levels and a slightly increased level of eotaxin-3 was identified after doxycycline treatment [124]. Clarithromycin was also demonstrated to contribute to the reduction of MMP-9 and TGF-β in an in vitro study; however, this drug did not exhibit any effect when assessed in a clinical setting [126]. Mepolizumab (an IL-5 antagonist), a recently emerging treatment option for recalcitrant type 2 CRS, was reported to reduce ECM protein deposition and decrease tissue eosinophil numbers [127,128,129]. Furthermore, TGF-β1 expression in airway eosinophils and decreased concentration of TGF-β1 in bronchoalveolar lavage fluid were also reduced by Mepolizumab treatment in asthma patients, suggesting the possible role of Mepolizumab in improving tissue remodeling in type 2 CRS [129].

In this review, we sought to discuss the tissue remodeling pattern and the accompanying immune responses that are induced by the inflammatory type of CRS. However, the underlying pathomechanism of tissue remodeling has yet to be elucidated and is, thus, being actively investigated. Efforts to more precisely classify CRS according to immune response and to elucidate the immune mechanisms are also being conducted to apply precision medicine in the management of CRS [130]. Future research should attempt to identify treatment targets to reverse tissue remodeling in CRS according to the underlying immune response.

## Figures and Tables

**Table 1:** Differences between Type 2 CRS and Non-type 2 CRS.

	Type 2	Non-Type 2
External stimuli	Allergen, *Staphylococcus aureus* biofilm/enterotoxin, and Fungi	Pollution and bacteria
Effector cells	Eosinophils, Th2 cells, ILC2s, B cells, basophils, and mast cells	Neutrophils, NK cells, Type 1: CD8+ T cells, Th1 cells, and ILC1s Type 3: Th17 cells and ILC3s
Primary cytokines	IL-4, IL-5, and IL-13	Type 1: IFN-γ and IL-12 Type 3: IL-17 and IL-22
Other mediators	IL-25, IL-33, TSLP, and IgE	IL-1β, IL-36, IL-6, IL-8, TGF-β, CXCL1, and CXCL10
Clinical features	Bilateral disease Ethmoid sinus > Maxillary sinus Headache/migraine, anosmia, nasal polyposis, asthma, aspirin-induced respiratory disease	Maxillary sinus > Ethmoid sinus Purulent nasal discharge
Tissue remodeling features	Barrier disruption, basal membrane thickening, and stromal edema	Basal membrane thickening, goblet cell hyperplasia, fibrosis, and collagen deposition

## Abbreviations

CRS	Chronic rhinosinusitis
AR	Allergic rhinitis
BMT	Basement membrane thickening
CCL	Chemokine (C-C motif) ligand
CRSsNP	Chronic rhinosinusitis without nasal polyp
CRSwNP	Chronic rhinosinusitis with nasal polyp
CXCL	Chemokine (C-X-C motif) ligand
ECM	Extracellular matrix
EMT	Epithelial-mesenchymal transition
ERK	Extracellular signal-regulated kinase
HDAC	Histone deacetylase
HIF-1α	Hypoxia-inducible factor -1α
IFN-γ	Interferon-γ
IgE	Immunoglobulin E
IL	Interleukin
ILC1s	Type 1 innate lymphoid cells
ILC2s	Type 2 Innate lymphoid cells
ILC3s	Type 3 innate lymphoid cells
MMP	Matrix metalloproteinase
MPO	Myeloperoxidase
MUC5AC	Mucin 5AC
MUC5B	Mucin 5B
OSM	Oncostatin
P-gp	P-glycoprotein
*RUNX2*	*Runt-related transcription factor 2*
*S.aureus*	*Staphylococcus aureus*
SIRT1	Sirtuin 1
TGF-β	Transforming growth factor-β
Th1	Type 1 T helper
Th17	Type 17 T helper
Th2	Type 2 T helper
TIMP	Tissue inhibitors of metalloproteinases
TNF-α	Tumor necrosis factor α
tPA	Tissue plasminogen activator
Tregs	Regulatory T cells
TSLP	Thymic stromal lymphopoietin
uPA	Urokinase-type plasminogen activator
α-SMA	Smooth muscle actin
